# Prolonged response to recombinant human erythropoietin treatment in patients with myelodysplastic syndrome at a single referral centre in Brazil

**DOI:** 10.6061/clinics/2019/e771

**Published:** 2019-09-04

**Authors:** Anna Thawanny Gadelha Moura, Fernando Barroso Duarte, Maritza Cavalcante Barbosa, Talyta Ellen de Jesus dos Santos, Romélia Pinheiro Gonçalves Lemes

**Affiliations:** I Laboratorio de Pesquisa em Hemoglobinopatias e Genetica das Doencas Hematologicas, Universidade Federal do Ceara, Fortaleza, CE, BR; II Departamento de Cirurgia, Universidade Federal do Ceara, Fortaleza, CE, BR

**Keywords:** Myelodysplastic Syndrome, Recombinant Human Erythropoietin, Erythropoietin, Epoetin Alfa, EPO, EPO Alfa

## Abstract

**OBJECTIVES:**

To evaluate the effects of epoetin (EPO) alfa treatment on overall survival, event-free survival and response duration in patients with myelodysplastic syndrome (MDS) who were treated at a haematological referral centre in northeastern Brazil.

**METHODS:**

This was a retrospective cohort study of 36 patients diagnosed with MDS and treated with EPO alfa at 30,000 to 60,000 IU per week. Clinical data were collected from medical records. The events assessed were non-response to treatment and progression to acute myeloid leukaemia (AML). Statistical analyses were performed using GraphPad Prism 7 and SPSS 24 software.

**RESULTS:**

The overall survival of patients who received EPO alfa treatment was 51.64%, with a median of 65 months of treatment, and the overall survival of this group was 100% during the first 24 months. We detected a 43.5-month median event-free survival, with a response rate of 80.5%. We observed responses from 25 to 175 months. Patients with transfusion dependence and those with a high-risk stratification, as determined by the International Prognostic Scoring System (IPSS), the Revised International Prognostic Scoring System (IPSS-R), the WHO classification-based Prognostic Scoring System (WPSS) and the WHO 2016, had a lower event-free survival than other patients.

**CONCLUSIONS:**

Despite the wide use of EPO alfa in the treatment of anaemia in patients with MDS, the median response duration is approximately only 24 months. Our data provide encouraging results concerning the benefits of using EPO alfa for the improvement of the quality of life, as patients treated with EPO showed higher overall survival, event-free survival rates and longer response durations than have been previously described in the literature.

## INTRODUCTION

Myelodysplastic syndrome (MDS) is a clonal haematopoietic disease characterized by ineffective haematopoiesis, dysplasias, peripheral cytopenias, and the risk of transformation into acute myeloid leukaemia (AML) 1. Approximately 60% of patients with MDS have severe anaemia at diagnosis, and this finding has been frequently associated with an impaired quality of life, reduced motor and cognitive functions, low haemoglobin concentrations, and transfusion dependence 2.

Erythropoiesis-stimulating agents (ESAs), such as recombinant human erythropoietin (EPO), have been used to treat anaemia in patients with MDS; ESAs constitute an important therapeutic alternative since they reduce transfusion requirements and the risks associated with transfusions 3. Several studies have shown a greater overall survival of MDS patients treated with ESAs than untreated anaemic patients 4,5, and ESAs have been demonstrated to be efficacious and to have a safety profile characterized by few adverse events 6. Different isoforms of EPO are available; these isoforms differ in their structure and response strength 7,8.

The refractoriness rate for ESAs is approximately 40-50% after 2 years of treatment 4. The effectiveness of ESA treatment seems to be associated with some clinical characteristics, such as low serum EPO levels (<500 IU/L), a lack of transfusion dependence, and a low-risk stratification based on the Revised International Prognostic Scoring System (IPSS-R) 3,9,10,11. However, the mechanisms that drive this refractoriness have not been clarified.

Thus, the aim of the present study was to evaluate the effects of epoetin (EPO) alfa treatment on the event-free survival and response duration of patients with MDS who were treated at a haematological referral centre in northeastern Brazil.

## MATERIALS AND METHODS

This was a retrospective cohort study of 36 adult patients of both genders who were diagnosed with MDS and treated with EPO alfa. Follow-up occurred at the Haematology Department of the Hospital Universitário Walter Cantídeo (HUWC), Ceará, Brazil.

MDS was diagnosed according to the minimum criteria established at the Vienna Conference on MDS in 2006 12. Patients were stratified according to their risk of progression to AML, according to the criteria of the World Health Organization 2016 (WHO 2016), the International Prognostic Scoring System (IPSS), the IPSS-R, and the WHO Classification-based Prognostic Scoring System (WPSS).

The response to treatment with EPO alfa was assessed according to the criteria established by the International Working Group (IWG) in 2006. The IWG defines a response as an increase in haemoglobin levels of 1.5 g/dL or a reduction in the transfusion requirement of four units after every 8 weeks of treatment 13.

Clinical data related to age, sex, blood count, treatment time, EPO alfa dose, transfusion dependence, myelogram, bone biopsy, and karyotype were collected from medical records.

Of the 42 patients treated with EPO alfa, six patients (14.3%) who did not have medical records available before treatment (which precluded any analysis of the evolution of the response) were excluded from the study.

Statistical analyses were performed using GraphPad Prism software version 7.0 and SPSS software version 24. The results are expressed as the mean ± standard error of the mean (SEM). A bivariate analysis was performed using the chi-square test, and the global survival and event-free survival analyses were performed using Kaplan-Meier curves. The events considered for the analysis were non-response to EPO alfa treatment and progression to AML. Values of *p*<0.05 were considered statistically significant.

## RESULTS

The study population had a median age of 75 years (49-95 years) and had a predominance of females (55.55% female and 44.45% male). Most patients were classified as low/very low risk according to the IPSS-R (72.2%) and the WPSS (83.3%). Based on the WHO classification, the majority of patients were classified as MDS with multilineage dysplasia (MDS-MLD) (38.9%), followed by MDS with ringed sideroblasts (MDS-RS) (22.2%), MDS with dysplasia (MDS-SLD) (13.9%), MDS with excess blasts -1,2 (MDS-EB-1,2) (8.3%), and MDS with del (5q) isolated (11.1%); additionally, two patients (5.6%) progressed to AML (Table 1).

**Figure 1:**
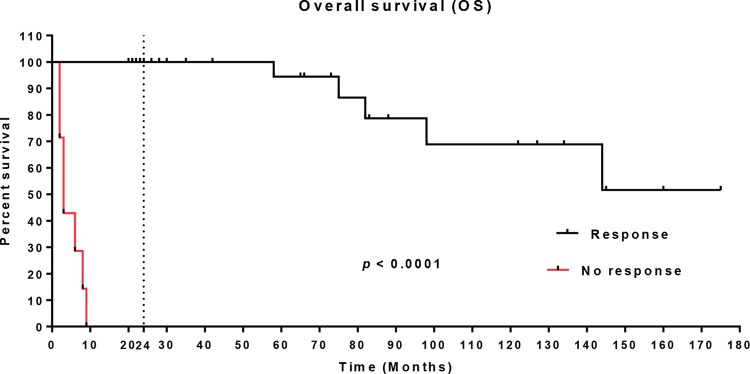
Overall survival (OS) of the study population treated with epoetin alfa.

The median follow-up was 50 months, ranging from 2 to 175 months. The overall survival of patients who had a sustained response to EPO alfa treatment was 51.64%, with a median of 65 months of treatment. During the first 24 months, the overall survival of this group was 100%. For the group of patients who were refractory to EPO alfa treatment, the overall survival was 14.28%, with a median of 3 months, which was significantly lower than the survival of the group who responded to EPO alfa (*p*<0.0001) (Figure 1).

**Figure 2:**
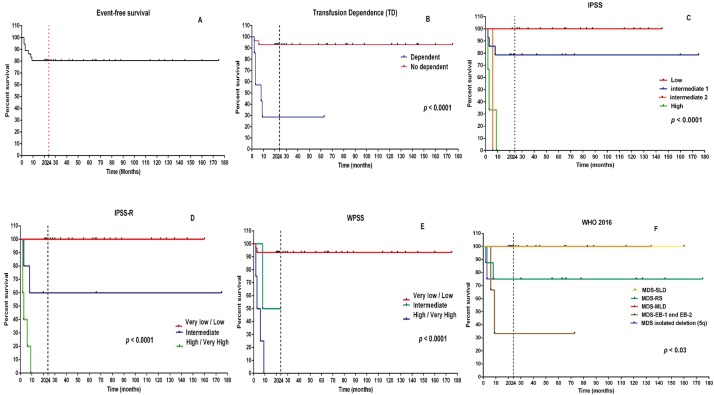
Survival analyses in the study population. (A) Event-free survival of the EPO alfa treated population. (B) Influence of transfusion dependence on event-free survival. (C-F) Influence of risk stratification on event-free survival.

**Figure 3:**
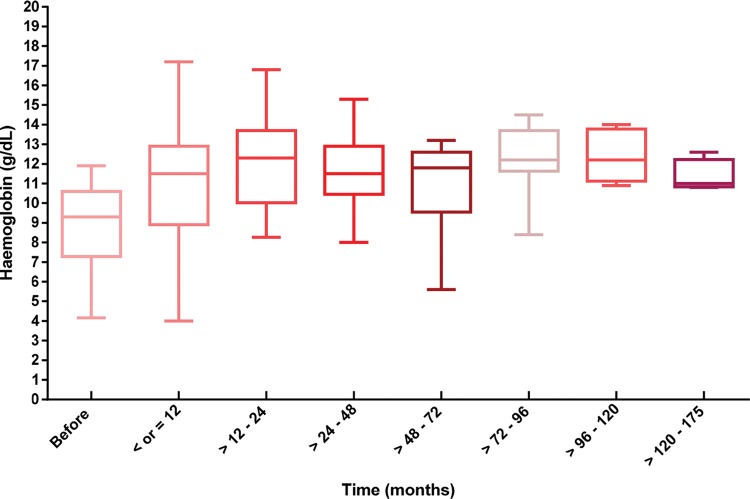
The mean haemoglobin of the population studied during treatment with EPO alfa.

Event-free survival throughout the follow-up period was 80.5% (Figure 2A and Table 1). Haemoglobin values increased significantly after 24 months and up to 175 months after the initiation of EPO alfa treatment, and the haemoglobin values remained higher than the values at the initiation of treatment (*p*<0.001) (Figure 3).

Patients with transfusion dependence (*p*<0.0001), those classified as high risk based on the IPSS (*p*<0.0001) and those classified as high/very high based on the IPSS-R (*p*<0.0001) and the WPSS (*p*=0.0005) had lower event-free survival (Figure 2B-2E) than the other patients.

The WHO classification was also able to stratify groups based on the response to treatment with EPO alfa. Event-free survival rates were lower in patients classified as MDS-RS, MDS-EB and del 5q than in patients classified as MDS-SLD and MDS-MLD (*p*=0.03) (Figure 2F).

After up to 175 months of treatment with EPO alfa, the event-free survival rates were greater than 93.3% in patients with a low/very low-risk stratification and no transfusion dependency (Figure 2B-2E).

A significant association was observed among the chi-square test for karyotype (very poor, poor, intermediate, and good, *p*=0.0015); normal, complex, trisomy, with del 5q and monosomy (*p*=0.0205); and the response to EPO alfa treatment (yes, no) (Table 1).

The main findings from the cytogenetic analysis showed normal karyotypes in 77.77% (28/36) of patients. Patients with a normal karyotype showed an 89.3% (25/28) response to EPO alfa treatment; in contrast, there was a 50% (4/8) response rate in patients with an altered karyotype. Only 10.75% (3/28) of the patients with a normal karyotype did not respond to treatment with EPO alfa. None of the patients with a normal karyotype were transfusion dependent (Table 1).

Patients with a good karyotype were more common (88.9%, 32/36) than patients in the other cytogenetic risk groups of the IPSS-R. Only 15.6% of the patients with a good karyotype (5/32) were transfusion dependent, and 60% (3/5) of these patients did not respond to EPO alfa. Seventy-five percent of the patients with a 5q deletion responded to treatment (Table 1).

The poor and very poor cytogenetic risk groups (5.55%, 2/36), which corresponded to complex karyotypes, did not respond to EPO alfa (0%). The intermediate risk group (5.55%, 2/36) included one patient with a transfusion-dependent trisomy of chromosome 8, who also did not respond to EPO alfa treatment, and one patient with a monosomy of chromosome 18, who showed a non-transfusion-dependent response to treatment.

Only 33.33% (12/36) of patients had serum erythropoietin (sEPO) level data available in their medical records: 16.66% had sEPO levels >200 mU/mL, and 83.33% had levels <200 mU/mL. Patients who had elevated sEPO levels did not respond to treatment (100%). Most of the patients with sEPO levels <500 mU/mL (83.3%, 10/12) showed a response to EPO alfa. None of the patients had serum erythropoietin values >500 mU/mL (Table 1).

Most patients (91.66%, 33/36) started with low doses (30,000 IU) of EPO alfa that were administered weekly, and most patients showed a positive response to treatment (78.78%, 26/33). Only one patient (33.33%, 1/3), who presented with a trisomy of chromosome 8, did not respond to high initial EPO alfa doses (60,000 IU) (Table 1). No statistically significant association was found between the response to treatment and the initial dose of EPO alfa (*p*=0.5412)

Some patients (82.75% 24/29) had a notable and surprisingly prolonged response, ranging from 25 to 175 months. All of these patients were transfusion independent, and the majority of these patients were classified by the WHO 2016 and IPSS-R as having a favourable response stratification. Most patients (87.5%) had blasts ≤2%. Patients with a normal karyotype were predominant (83.3%). Only one patient had a karyotype with monosomy 18 (4.1%), and three karyotypes showed a 5q- deletion (12.5%). Most patients (95.8%) also had high platelet levels (≥100,000/mm3) (Table 2).

## DISCUSSION

Some multicentre studies that assessed the treatment of MDS patients with EPO reported response rates of approximately 30-50%, with a duration of 12-24 months 4,5,14,15. In contrast, the patients from our study centre had a higher response rate and a longer duration response.

The results of two recent prospective, randomized, placebo-controlled trials of ESAs in MDS patients with anaemia 16,17 were compared with our data. For example, the ARCADE 16 study showed a significantly lower incidence of transfusions during treatment in patients receiving darbepoetin alfa than in those receiving a placebo (*p*=0.008), and the darbepoetin-treated patients showed increased erythroid response rates (14.7%; *p*=0.016). This erythroid response rate was comparable to that reported for the EPOANE 3021 trial 17, where 31.8% of the patients treated with EPO alfa reached the target response erythroid level, whereas 4.4% of the placebo-treated patients reached the target response level (*p*<0.001). In our study, none of the transfusion-independent patients required transfusions during treatment with EPO, and transfusion dependence was also reduced. These results are particularly significant for everyday clinical practice, where the goal in the management of patients with low-risk MDS is to achieve transfusion independence, as transfusion independence is associated with improved survival 18. Our sustained rates of erythroid response following the initiation of EPO alfa treatment may also reflect the need for prolonged treatment to obtain the full clinical benefit.

Another interesting finding was related to the incidence of elevated haemoglobin levels, which was lower than expected in the EPOANE 3021 and ARCADE studies. The suggested cause for this result was that a dose adjustment above the initial low-dose regimen was allowed only after 8 weeks in the EPOANE 3021 study and after 31 weeks in the ARCADE study. In addition, a higher response rate was observed in patients treated with higher doses of ESAs (80,000 IU/week). Other studies on ESA treatments have reported the use of significantly higher doses of EPO alfa, ranging from 40,000-80,000 IU/week, in patients with MDS than in patients with other clinical indications, as higher dosing is thought to aid in counteracting the intrinsic resistance of MDS erythroid precursors to EPO alfa 19,20,21. In the present study, the administered dose range was 30,000-60,000 IU/week 22, and dose adjustments were based on the patient haemoglobin levels (a reduction or increase of 1.5 g/dL of haemoglobin in 30 days in the absence/presence of erythrocyte transfusions) and clinical status. Doses were adjusted for 97.22% (35/36) of the patients. In patients with MDS, when a positive response is obtained, ESA doses can be adjusted to the lowest effective dose that maintains haemoglobin levels within the normal range. Thus, a high percentage of patients with low-risk MDS will respond to treatment with EPO alfa and have higher response rates 23.

Another noteworthy observation was that no patients received hypomethylating agents (azacitidine or decitabine) and lenalidomide in addition to EPO alfa. Some studies have suggested that erythroid response rates can be increased with this type of combination therapy 24,25.

High-risk stratifications and transfusion dependence had a negative impact on event-free survival in the patients in this study and could be considered predictors of a poor response to EPO alfa treatment. These findings agree with previous studies that observed lower rates and shorter durations of the response to EPO treatment in patients at high risk and with transfusion dependence 26.

The WHO stratification is an important tool for determining the most appropriate therapeutic management for each patient. Published studies have shown that MDS-RS, MDS-EB, and del (5q) patients have a better response to ESA therapy, whereas the MDS-SLD and MDS-MLD groups have a worse response, regardless of transfusion dependence. Not much evidence is available and published on this topic, but patients with a 5q chromosomal abnormality seem to respond with a significantly lower response rate to both the EPO alfa and darbepoetin isoforms (with or without G-CSF) than MDS patients without a 5q deletion 27. This finding is in agreement with our data, which also revealed a low response rate to EPO alfa in subjects with a 5q deletion. Although three patients with 5q deletions presented prolonged responses, the response rates were low.

The characteristics of the patients who had significantly longer responses to treatment (25 to 154 months) confirmed the favourable prognosis and response of patients in the low-risk categories, which are characterized by no transfusion dependence, a normal karyotype, and a small number of bone marrow blasts 28. In patients with MDS, prognosis was better in those with a normal karyotype than in those with the presence of any structural or numerical changes. The karyotype also plays an important role in establishing the MDS diagnosis because during the course of the disease, cytogenetic methods are used to assess the response to therapy or to examine clonal evolution, which is a sign of disease progression 29.

Among the patients who did not respond to treatment, the predominant features included a complex karyotype, trisomy 8 and transfusion dependence. Patients with a complex karyotype showed an accumulation of progressive changes and greater disease aggressiveness, which were associated with an extremely unfavourable prognosis and a high propensity to transform into acute leukaemia within a short period of time. This observation was confirmed as a statistically significant association in our study (*p*=0.01), thereby demonstrating the importance of cytogenetic studies in the evolutionary monitoring of MDS. Patients with trisomy 8, as an isolated anomaly, have a significantly higher risk of leukaemic transformation than other patients, but the significance of the gain of an extra chromosome 8 is not yet fully understood 29,30.

In recent years, the value of serum erythropoietin in MDS has been explored in the literature as a predictor of the response to EPO. The role of sEPO levels as a prognostic risk factor in MDS also remains poorly understood. Despite the benefits of growth factor therapy, and of erythropoietin therapy in particular, studies have shown that sEPO levels (<500 IU/L) are predictive of response 9,10,11. Patients who present with low sEPO levels associated with anaemia before treatment appear to respond more readily to EPO. These observations are further supported by the findings of other EPO studies 14,31. We observed a response in greater than 80% of patients with low sEPO levels. A limitation of the present study was the number of patients and the lack of availability of data on the sEPO levels in all patients; however, this examination is not covered by the Brazilian Unified Health System (SUS).

The results obtained in the present study support the importance of EPO alfa treatment for patients with MDS and suggest that the use of EPO alfa might achieve durable responses, thereby guaranteeing an improvement in the quality of life of these patients. However, the evaluation of each patient's clinical characteristics, such as their risk stratification and transfusion dependence, is essential prior to the initiation of EPO alfa treatment.

## AUTHOR CONTRIBUTIONS

Moura ATG was responsible for planning the study, collecting data from medical records, building the database, analysing the data, performing statistical analysis and drafting the manuscript. Duarte FB assisted with the planning of the study and the revision of the manuscript. Santos TEJ revised the manuscript. Lemes RPG assisted with the planning of the study and the revision of the manuscript. Barbosa MC assisted with the statistical analysis and with the final manuscript drafting.

## Figures and Tables

**Table 1:** Response to EPO alfa treatment according to the clinical-laboratory characteristics of the study population (n=36).

Variable	Subgroup	Response		X^2^* (*p* value)
		Yes	No	
		n/n total (%)	n/n total (%)	
		29 / 36 (80.5%)	7/ 36 (19.44%)	
**SOCIO-DEMOGRAPHIC VARIABLES**				
Gender	Female	16 / 20 (80%)	4 / 20 (20%)	1
	Male	13 / 16 (81.2%)	3 / 16 (18.8%)	
Age	<75	11 / 15 (73.3%)	4 / 15 (26.7%)	0.4178
	≥75	18 / 21 (85.7%)	3 / 21 (14.3%)	
	≤60	1 / 1 (100%)	0 / 1 (0%)	0.6502
	>60 - 75	13 / 17 (76.5%)	4 / 17 (23.5%)	
	>75 - 90	13 / 16 (81.2%)	3 / 16 (18.8%)	
	>90	2 / 2 (100%)	0 / 2 (0%)	
**BONE MARROW FINDINGS**				
Cellularity of the bone marrow	Hypocellular	6 / 9 (66.7%)	3 / 9 (33.3%)	0.1855
	Normocellular	9 / 9 (100%)	0 / 9 (0%)	
	Hypercellular	14 / 18 (77.8%)	4 / 18 (22.2%)	
Ring sideroblasts	Presence	21 / 24 (87.5%)	4 / 24 (16.7%)	0.3945
	Absence	8 / 12 (66.7%)	4 / 12 (33.3%)	
% Bone marrow blast	≤2%	25 / 26 (96.1%)	1 / 26 (3.9%)	<0.0001
	>2% - <5%	3 / 4 (75%)	1 / 4 (25%)	
	5% - 10%	1 / 3 (33.3%)	2 / 3 (66.7%)	
	>10%	0 / 3 (0%)	3 / 3 (100%)	
**PERIPHERAL BLOOD FINDINGS**				
Haemoglobin IPSS-R (g/dL)*	≥10	11 / 12 (91.7%)	1 / 12 (8.3)	
	8 - 10	11 /13 (84.6%)	2 / 13 (15.4%)	0.0922
	<8	7 / 11 (63.6%)	4 / 11 (36.4%)	
ANC IPSS-R^‡^	<0.8	6 / 7 (85.7%)	1 / 7 (14.3%)	1
	≥0.8	23 / 29 (79.3%)	6 / 29 (20.7%)	
Platelets IPSS-R^†^	≥50	2 / 4 (50%)	2 / 4 (50%)	
	>50 - 100	1 / 3 (33.3%)	2 / 3 (66.7%)	0.0136
	>100	26 / 29 (89.7%)	3 / 29 (10.3%)	
Cytopenias^§^	0 - 1	16 / 17 (94.1%)	1 / 17 (5.9%)	0.0918
	2 - 3	13 / 19 (68.4%)	6 / 19 (31.6%)	
**CYTOGENETIC CHARACTERIZATION AND PROGNOSTIC IMPACT**				
Karyotype category 1	Normal	25 / 28 (89.3%)	3 / 28 (10.7%)	0.0301
	Changed	4 / 8 (50%)	4 / 8 (50%)	
Karyotype IPSS-R	Very poor	0 / 1 (0%)	1 / 1 (100%)	
	Poor	0 / 1 (0%)	1 / 1 (100%)	0.0015
	Intermediary	1 / 2 (50%)	1 / 2 (50%)	
	Good	28 / 32 (87.5%)	4 / 32 (12.5%)	
Karyotype category 2	Normal	25 / 28 (89.3%)	3 / 28% (10.7%)	
	Del.5q	3 / 4 (75%)	1 / 4 (25%)	0.0205
	Complex	0 / 2 (0%)	2 / 2 (100%)	
	Trisomy	0 / 1 (0%)	1 / 1 (100%)	
	Monosomy	1 / 1 (100%)	0 / 1 (0%)	
Risk group IPSS-R	Very low	10 / 10 (100%)	0 / 10 (0%)	<0.0001
	Low	16 / 16 (100%)	0 / 16 (0%)	
	Intermediary	3 / 5 (60%)	2 / 5 (40%)	
	High	0 / 4 (0%)	4 / 4 (100%)	
	Very high	0 / 1 (0%)	1 / 1 (100%)	
Risk group WPSS	Very low	12 / 13 (92.3%)	1 / 13 (7.7%)	<0.0001
	Low	16 / 17 (94.1%)	1 / 17 (5.9%)	
	Intermediate	1 / 1 (50%)	1 / 1 (50%)	
	High	0 / 4 (0%)	4 / 4 (100%)	
Risk group IPSS	Low	18 / 18 (100%)	0 / 18 (0%)	<0.0001
	Intermediary 1	11/14 (78.6%)	3 / 14 (21.4%)	
	Intermediary 2	0 / 1 (0%)	1 / 1 (100%)	
	High	0 / 3 (0%)	3 / 3 (100%)	
WHO 2016	MDS-SLD	5 / 5 (100%)	0 / 5 (0%)	0.0083
	MDS-RS	6 / 8 (75%)	2 / 8 (25%)	
	MDS-MLD	14 / 14 (100%)	0 / 14 (0%)	
	MDS-EB-1	0 / 1 (0%)	1 / 1 (100%)	
	MDS-EB-2	1 / 2 (50%)	1 / 2 (50%)	
	MDS with isolate (5q)	3 / 4 (75%)	1 / 4 (25%)	
	Evolution to AML	0 / 2 (0%)	2 / 2 (100%)	
Transfusion dependence	Yes	2 / 7 (28.6%)	5 / 7 (71.6%)	0.001
	No	27 / 29 (93.1%)	2 / 29 (6.9%)	
Serum erythropoietin (mU/mL)^∥^	<500	10 /12 (83.3%)	2 / 12 (16.7%)	
	>500	0 / 0 (0%)	0 / 0 (0%)	
	<200	9 / 10 (90%)	1 / 10 (10%)	
	>200	0 / 2 (0%)	2 / 2 (100%)	
Initial dose EPO alfa UI/week	30,000	27 / 33 (81.8%)	6 / 33 (18.2%)	0.4882
	60,000	2 / 3 (66.7%)	1 / 3 (33.3%)	
**RESPONSE TO TREATMENT WITH EPO ALFA**	Months	Event-free survival (%)		
	8	83.3		
	24	80.5		
	48	80.5		
	60	80.5		
	150	80.5		
**MEAN HAEMOGLOBIN (G/DL)**	Treatment time (months)	mean ± SD		
	Before	8.89 ± 2.083		
	≤12	10.87 ± 3.063		
	>12-24	12.09 ± 2.220		
	>24-48	11.64 ± 1.766		
	>48-72	10.85 ± 2.472		
	>72-96	12.29 ± 1.708		
	>96-120	12.36 ± 1.168		
	>120-175	11.35 ± 0.652		

Data are n (%) unless indicated otherwise. The relevant cut points were:

* hemoglobin values of < 8 g/dL, 8 - < 10 g/dL, and ≥10 g/dL;

† platelet values of < 50 × 10^9^/L, 50 100 × 10^9^/L and ≥ 100 × 10^9^/L, and

‡ absolute neutrophil counts (ANC) of < 0.8 × 10^9^/L *versus* ≥ 0.8 × 10^9^/L.

§ Cytopenias were defined as hemoglobin < 11 g/dL, absolute neutrophil count < 0.8 × 10^9^/L or platelets < 100 × 10^9^/L.

∥ Not all patients had data available, so percentages are based on twelve patients (n=12). Abbreviations: World Health Organization 2016 (WHO 2016); International Prognostic Scoring System (IPSS); Revised International Prognostic Scoring System (IPSS-R); the WHO classification-based Prognostic Scoring System (WPSS); absolute neutrophil count (ANC); and Standard deviation (SD).

**Table 2:** Patients with a response of greater than 24 months (25 to 175 months, n=24).

	Age	Karyotype IPSS-R	Karyotype	Transfusion dependence	IPSS-R	WHO 2016	% Blasts MO	Haemoglobin IPSS-R*	Platelets IPSS-R^†^	Response time to EPO alfa (months)
1	64	Good	Normal	No	Very low	MDS-MLD	≤2%	≥10	≥100	28
2	95	Good	deletion 5q-	No	Very low	MDS with isolated del 5q-	≤2%	≥10	≥100	82
3	94	Good	Normal	No	Very low	MDS-RS	≤2%	≥10	≥100	145
4	73	Good	Normal	No	Low	MDS-MLD	≤2%	<8	≥100	42
5	70	Good	Normal	No	Low	MDS-MLD	≤2%	8-10	≥100	134
6	75	Good	deletion 5q-	No	Low	MDS with isolated del 5q-	≤2%	<8	≥100	75
7	75	Good	Normal	No	Low	MDS-RS	≤2%	8-10	≥100	30
8	49	Good	Normal	No	Low	MDS-MLD	≤2%	8-10	≥100	66
9	76	Good	Normal	No	Very low	MDS-SLD	≤2%	8-10	≥100	83
10	82	Good	Normal	No	Very low	MDS-MLD	≤2%	≥10	≥100	144
11	82	Good	Normal	No	Low	MDS-EB-2	5%-10%	8-10	≥100	73
12	65	Good	Normal	No	Very low	MDS-MLD	≤2%	≥10	≥100	26
13	74	Good	Normal	No	Low	MDS-RS	≤2%	≥10	<50	127
14	65	Good	Normal	No	Very low	MDS-MLD	≤2%	≥10	≥100	26
15	70	Good	Normal	No	Intermediary	MDS-RS	>2% - <5%	<8	≥100	175
16	75	Good	Normal	No	Very low	MDS-MLD	≤2%	≥10	≥100	88
17	86	Good	Normal	No	Very low	MDS-MLD	≤2%	≥10	≥100	65
18	82	Good	Deletion 5q-	No	Very low	MDS with isolated del 5q	≤2%	≥10	≥100	66
19	68	Good	Normal	No	Low	MDS-MLD	≤2%	8-10	≥100	58
20	73	Good	Normal	No	Intermediary	MDS-MLD	≤2%	<8	≥100	66
21	86	Good	Normal	No	Low	MDS-MLD	>2% - <5%	≥10	≥100	35
22	61	Intermediary	Monosomy	No	Low	MDS-SLD	≤2%	8-10	≥100	160
23	80	Good	Normal	No	Low	MDS-RS	≤2%	8-10	≥100	122
24	81	Good	Normal	No	Low	MDS-RS	≤2%	8-10	≥100	98

* Hemoglobin values of < 8 g/dL, 8 - < 10 g/dL and ≥ 10 g/dL;

† platelet values of < 50 × 10^9^/L, 50-100 × 10^9^/L and ≥ 100 × 10^9^/L;

^§^Cytopenias were defined as hemoglobin < 11 g/dL; absolute neutrophil count < 0.8 × 10^9^/L; or platelets < 100 × 10^9^/L. Abbreviations: Revised International Prognostic Scoring System (IPSS-R); World Health Organization 2016 (WHO 2016); MDS with multidrug dysplasia (MDS-MLD), MDS with ringed sideroblasts (MDS-RS), MDS with dysplasia (MDS-SLD), MDS with excess blasts 2 (MDS-EB-2), and MDS with del (5q) isolated.

## References

[1] Malcovati L, Hellström-Lindberg E, Bowen D, Adàs L, Cermak J, Del Caãizo C (2013). Diagnosis and treatment of primary myelodysplastic syndromes in adults: recommendations from the European LeukemiaNet. Blood.

[2] Oliva EN, Finelli C, Santini V, Poloni A, Liso V, Cilloni D (2012). Quality of life and physicians’ perception in myelodysplastic syndromes. Am J Blood Res.

[3] Park S, Kelaidi C, Sapena R, Vassilieff D, Beyne‐Rauzy O, Coiteux V (2010). Early introduction of ESA in low risk MDS patients may delay the need for RBC transfusion: a retrospective analysis on 112 patients. Leuk Res.

[4] Park S, Grabar S, Kelaidi C, Beyne‐Rauzy O, Picard F, Bardet V (2008). Predictive factors of response and survival in myelodysplastic syndrome treated with erythropoietin and G‐CSF: the GFM experience. Blood.

[5] Jadersten M, Malcovati L, Dybedal I, Della Porta MG, Invernizzi R, Montgomery SM (2008). Erythropoietin and granulocyte-colony stimulating factor treatment associated with improved survival in myelodysplastic syndrome. J Clin Oncol.

[6] Kelaidi C, Fenaux P (2010). Darbepoetin alfa in anemia of myelodysplastic syndromes: present and beyond. Expert Opin Biol Ther.

[7] Egrie JC, Browne JK (2001). Development and characterization of novel erythropoiesis stimulating protein (NESP). Br J Cancer.

[8] Brinks V, Hawe A, Basmeleh AH, Joachin-Rodriguez L, Haselberg R, Somsen GW (2011). Quality of original and biosimilar epoetin products. Pharm Res.

[9] Stenke L, Wallvik J, Celsing F, Hast R (1993). Prediction of response to treatment with human recombinant erythropoietin in myelodysplastic syndromes. Leukemia.

[10] Santini V (2011). Clinical use of erythropoietic stimulating agents in myelodysplastic syndromes. Oncologist.

[11] Cortesão E, Tenreiro R, Ramos S, Pereira M, César P, Carda JP (2015). [Serum Erythropoietin as Prognostic Marker in Myelodysplastic Syndromes]. Acta Med Port.

[12] Valent P, Horny HP, Bennett JM, Fonatsch C, Germing U, Greenberg P (2007). Definitions and standards in the diagnosis and treatment of the myelodysplastic syndromes: Consensus statements and report from a working conference. Leuk Res.

[13] Cheson BD, Greenberg PL, Bennett JM, Lowenberg B, Wijermans PW, Nimer SD (2006). Clinical application and proposal for modification of the International Working Group (IWG) response criteria in myelodysplasia. Blood.

[14] Ross SD, Allen IE, Probst CA, Sercus B, Crean SM, Ranganathan G (2007). Efficacy and safety of erythropoiesis-stimulating proteins in myelodysplastic syndrome: a systematic review and meta-analysis. Oncologist.

[15] Kelaidi C, Beyne‐Rauzy O, Braun T, Sapena R, Cougoul P, Ades L (2013). High response rate and improved exercise capacity and quality of life with a new regimen of darbepoetin alfa with or without filgrastim in lower‐risk myelodysplastic syndromes: a phase II study by the GFM. Ann Hematol.

[16] Fenaux P, Santini V, Spiriti MA, Giagounidis A, Schlag R, Radinoff A (2016). Randomized, double-blind, placebo-controlled, multicenter study, evaluating Epoetin-alfa versus placebo in anemic patients with IPSS Low-Int1 risk MDS [abstract]. Haematologica.

[17] Platzbecker U, Symeonidis A, Oliva EN, Goede JS, Delforge M, Mayer J (2017). A phase 3 randomised placebo-controlled trial of darbepoetin alfa in patients with anemia and lower-risk myelodysplastic syndromes. Leukemia.

[18] Garelius HK, Johnston WT, Smith AG, Park S, de Swart L, Fenaux P (2017). Erythropoiesis‐stimulating agents significantly delay the onset of a regular transfusion need in nontransfused patients with lower‐risk myelodysplastic syndrome. J Intern Med.

[19] Musto P, Falcone A, Sanpaolo G, Bodenizza C, La Sala A, Perla G (2003). Efficacy of a single, weekly dose of recombinant erythropoietin in myelodysplastic syndromes. Br J Haematol.

[20] Cortelezzi A, Colombo G, Pellegrini C, Silvestris I, Moronetti Mazzeo L, Bosari S (2008). Bone marrow glycophorin-positive erythroid cells of myelodysplastic patients responding to high-dose rHuEPO therapy have a different gene expression pattern from those of nonresponders. Am J Hematol.

[21] Balleari E, Clavio M, Arboscello E, Bellodi A, Bruzzone A, Del Corso L (2011). Weekly standard doses of rh-EPO are highly effective for the treatment of anemic patients with low-intermediate 1 risk myelodysplastic syndromes. Leuk Res.

[22] National Comprehensive Cancer Network (NCCN) (2017). Clinical Practice Guidelines in Oncology: Myelodysplastic Syndromes. 2016. Version 1. https://www.nccn.org.

[23] Santini V (2015). Anemia as the main manifestation of myelodysplastic syndromes. Semin Hematol.

[24] Komrokji RS, Lancet JE, Swern AS, Chen N, Paleveda J, Lush R (2012). Combined treatment with lenalidomide and epoetin alfa in lower-risk patients with myelodysplastic syndrome. Blood.

[25] Itzykson R, Thepot S, Beyne-Rauzy O, Ame S, Isnard F, Dreyfus F (2012). Does addition of erythropoiesis stimulating agents improve the outcome of higher-risk myelodysplastic syndromes treated with azacitidine?. Leuk Res.

[26] Hellström-Lindberg E, Malcovati L (2008). Supportive care, growth factors, and new therapies in myelodysplastic syndromes. Blood Rev.

[27] Kelaidi C, Park S, Brechignac S, Mannone L, Vey N, Dombret H (2008). Treatment of myelodysplastic syndromes with 5q deletion before the lenalidomide era; the GFM experience with EPO and thalidomide. Leuk Res.

[28] Greenberg PL, Tuechler H, SchanZ J, Sanz G, Garcia-Manero G, Solé F (2012). Revised international prognostic scoring system for myelodysplastic syndromes. Blood.

[29] Bacher U, Schanz J, Braulke F, Haase D (2015). Rare cytogenetic abnormalities in myelodysplastic syndromes. Mediterr J Hematol Infect Dis.

[30] Chauffaille ML (2006). Chromosomal abnormalities in myelodysplastic syndrome. Rev Bras hematol hemoter.

[31] Kwon M, Ballesteros M, Perez I, Echeverria V, Calderon M, Patrignani N (2006). Unexpected response to erythropoietin therapy in intermediate-low IPSS myelodysplastic syndromes. Blood.

